# Light-dependent roles of the G-protein α subunit GNA1 of *Hypocrea jecorina *(anamorph *Trichoderma reesei*)

**DOI:** 10.1186/1741-7007-7-58

**Published:** 2009-09-03

**Authors:** Christian Seibel, Gabriela Gremel, Roberto do Nascimento Silva, André Schuster, Christian P Kubicek, Monika Schmoll

**Affiliations:** 1 Research Area Gene Technology and Applied Biochemistry, Institute of Chemical Engineering, Vienna University of Technology, Wien, Austria

## Abstract

**Background:**

The filamentous ascomycete *Hypocrea jecorina *(anamorph *Trichoderma reesei*) is primarily known for its efficient enzymatic machinery that it utilizes to decompose cellulosic substrates. Nevertheless, the nature and transmission of the signals initiating and modulating this machinery are largely unknown. Heterotrimeric G-protein signaling represents one of the best studied signal transduction pathways in fungi.

**Results:**

Analysis of the regulatory targets of the G-protein α subunit GNA1 in *H. jecorina *revealed a carbon source and light-dependent role in signal transduction. Deletion of *gna1 *led to significantly decreased biomass formation in darkness in submersed culture but had only minor effects on morphology and hyphal apical extension rates on solid medium. Cellulase gene transcription was abolished in Δ*gna1 *on cellulose in light and enhanced in darkness. However, analysis of strains expressing a constitutively activated GNA1 revealed that GNA1 does not transmit the essential inducing signal. Instead, it relates a modulating signal with light-dependent significance, since induction still required the presence of an inducer. We show that regulation of transcription and activity of GNA1 involves a carbon source-dependent feedback cycle. Additionally we found a function of GNA1 in hydrophobin regulation as well as effects on conidiation and tolerance of osmotic and oxidative stress.

**Conclusion:**

We conclude that GNA1 transmits a signal the physiological relevance of which is dependent on both the carbon source as well as the light status. The widespread consequences of mutations in GNA1 indicate a broad function of this Gα subunit in appropriation of intracellular resources to environmental (especially nutritional) conditions.

## Background

The saprophytic ascomycete *Hypocrea jecorina *(anamorph *Trichoderma reesei*) is well known as an industrial producer of cellulases. Its potential for secretion of high levels of cellulolytic enzymes has lead to a widespread application of the fungus in processes related to pulp and paper or textile industry [1-3]. Cellulase gene expression is inducer dependent and cellulose, lactose, sophorose and sorbitol have all been identified as carbon sources promoting cellulase gene expression. Conversely, glucose inhibits inducer uptake, while in the presence of glycerol induction of cellulase gene expression is still possible. The basal level of cellulase gene expression is carbon catabolite repressed on carbon sources allowing fast metabolism (such as hexoses and polyols) [4-6]. Several transacting proteins mediating this induction have been identified, including both activators (xylanase regulator 1 (XYR1), activator of cellulase expression 2 (ACE2), heme activation protein (HAP2/3/5) complex) as well as repressors (ACE1 and the carbon catabolite repressor protein 1 (CRE1)) [7-10]. The exact mechanisms involved in the cellulose to cellulase signal transduction network, however, remain poorly understood.

Recently, a connection between light response and carbon source signaling was discovered. One important factor in the respective regulatory mechanism was shown to be the PAS/LOV domain (PER-ARNDT-SIM (PAS) domain of the light, oxygen and voltage type) protein ENVOY [11]. ENVOY not only regulates *H. jecorina *light response but also influences carbon source signaling and impacts light-dependent regulation of numerous genes in various ways [11,12]. The function of ENVOY, with respect to cellulase expression, is both light and inducer-dependent, thus interlinking the two pathways. This connection led us to include light related signaling phenomena in our experiments in conjunction with cellulase gene expression and to investigate transcription under strictly controlled light conditions. In the following we could further show that the G-protein α subunit GNA3 is involved in regulation of cellulase gene expression in light [13]. The significant regulatory impact of constitutive activation of GNA3 on cellulase gene expression (10-fold upregulation in light) suggests the transmission of an important signal. Nevertheless, the fact that constitutive activation of GNA3 does not lead to inducer independent cellulase formation rules out that GNA3 transmits the crucial signal for the presence of cellulose.

Signal transduction via heterotrimeric G-proteins has been studied in numerous fungi and is now recognized as one of the most important signaling pathways. This pathway regulates fundamental biological functions such as growth and conidiation, sexual development, virulence, tolerance against various forms of stress and secondary metabolite production [14-18]. In recent years, G-protein α subunits have been cloned and characterized from a wide array of fungi [19], demonstrating considerable conservation of these proteins. Although it might thus be concluded that their function in important physiological processes should also be similar, very different phenotypic consequences were shown as a result of disrupting genes encoding these highly conserved fungal G-protein subunits. For example, despite high similarity at the amino acid level between *Cryphonectria parasitica *CPG-1 and *Neurospora crassa *GNA-1, deletion of CPG-1 abolished asexual sporulation [20], while deletion of GNA-1 did not influence conidiation [21]. Therefore, the specific role of a given Gα subunit can hardly be predicted by extrapolating results obtained with another species, especially if the natural habitats and lifestyles are different. However, most of the work available on heterotrimeric G-protein signaling was not performed under controlled light conditions. The fact that we found considerable differences between results upon growth in light as compared to darkness for the regulatory role of GNA3 (that is, enhanced cellulase gene transcription only in light) [13], indicates that this environmental cue is of crucial importance for signal transduction through this pathway in fungi.

Gα protein subunits can be classified into three major subgroups according to their similarity to mammalian orthologs: subgroup I, which inhibits adenylate cyclase and which is related to the mammalian Gαi-proteins; subgroup II, which has no homology with mammalian G-proteins; and subgroup III, which reportedly activates adenylate cyclase in most fungi and is related to mammalian Gαs-proteins [14]. Nevertheless there are also exceptions to that classification [22].

Both deletion as well as constitutive activation of Gα subunits are considered to result in increased abundance of free Gβ and γ subunits. Opposite effects of such strains, as would be expected, represent a function of the respective Gα subunit, whereas similar phenotypic traits caused by deletion or constitutive activation of a Gα subunit are interpreted as an indication for regulation by the Gβγ-proteins [23-25].

In this study we elucidate the roles of the *H. jecorina *subgroup I Gα subunit GNA1 in different physiological processes. Some of the important functions of GNA1 orthologs in other fungi are regulation of cellulase and hydrophobin gene expression in *C. parasitica *[24,26] and involvement in stress response and biomass accumulation in *N. crassa *[21,25]. The genome of *H. jecorina *comprises three Gα subunits [27], one of which (GNA3) has already been shown to be involved in light-dependent regulation of cellulase gene expression [13], but does not transmit the specific cellulose signal. Therefore, and because of the function of the *C. parasitica *Gα subunit CPG-1 in cellulase gene expression [26], we investigated whether the Gα subunit GNA1 would be responsible for this signal. We show that on cellulose, the Gα subunit GNA1 also has a light-dependent role in regulation of cellulase and hydrophobin gene transcription. Moreover we found hints as to a carbon source-dependent feedback regulation of *gna1 *transcription upon activation of GNA1. Although not a direct sensor for the presence of cellulose (which should be able to induce cellulase gene expression even in the absence of an inducer) GNA1 plays an important role in transmission of a carbon source signal, which has different effects in light and darkness. Besides these regulatory functions we also detected a role of GNA1 in regulation of growth and stress tolerance.

## Results

### Cloning and characterization of *H. jecorina gna1*

**Figure 1:**
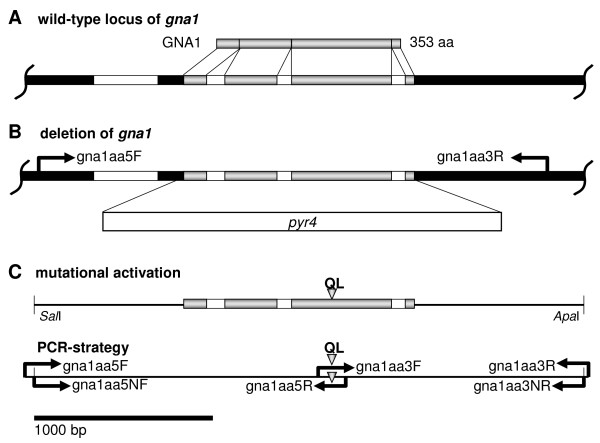
**Gene structure of *gna1*, deletion cassette and construct for transformation with the *gna1 *Q204L allele**. **(a) **The gene structure of *gna1 *is shown along with its exons and introns and the predicted GNA1 protein. **(b) **Construction of a transformation cassette for deletion of *gna1 *by replacing its open reading frame with the *pyr4 *gene as a selection marker. **(c) **Strategy for mutational activation of GNA1 by construction of a transformation cassette comprising a single amino acid exchange at position 204 as indicated by a triangle. The restriction sites *Sal*I and *Apa*I have been introduced using nested primers to facilitate cloning.

Searching the *T. reesei *genome database v2.0 [28] for proteins with high similarity to the *C. parasitica *Gα subunit CPG-1, which had been implicated in cellulase gene regulation [26], revealed the presence of a single copy of a gene encoding a class I Gα subunit, which consists of a predicted 1,296 bp open reading frame. Comparison of the cDNA and genomic sequences confirmed three introns within the open reading frame as indicated in the genome database and revealed a further, particularly long intron in the 5' untranslated region (Figure 1a). Following the guidelines set for the annotation of the *T. reesei *genome, we named this gene *gna1 *according to the already characterized ortholog of *N. crassa*. The deduced amino acid sequence of *H. jecorina *GNA1 corresponds to a protein of 353 amino acids with a calculated molecular mass of 40.9 kDa and contains a consensus site for *N*-myristoylation (MGXXXS) in the N-terminal region [29] as well as a consensus site for ADP ribosylation by pertussis toxin (CAAX) at the C-terminus [30] hence characterizing GNA1 as a typical member of the highly conserved class I G-protein α subgroup. The amino acid sequence of GNA1 shows 99% identity to *Hypocrea atroviridis *(*Trichoderma atroviride*) TGA1 (GenBank accession number AK74191). Moreover, GNA1 shows substantial similarity to *Gibberella zeae *GBA1 (EAA75079, 99%), *C. parasitica *CPC-1 (Q00580, 98%), *N. crassa *GNA-1 (AAA02560, 97%) and *Aspergillus fumigatus *GpaA/FadA (EAL90646.1; 94%).

Within 1.5 kb upstream of the *gna1 *translational start codon, which corresponds to an optimal Kozak consensus [31], several potential 5' upstream binding sites of transactivators are located: We found a GATA box (-1,495), a CCAAT box (-551, just upstream of the intron) and 11 CRE1-binding sites (5'-SYGGRG-3') in this genomic area. Furthermore, 3 stress response elements (5'-AGGGG-3'; -157, -477 and -875), [32,33] 2 of which were located within the large 5' untranslated region (UTR) intron, were detected. Of these stress response elements the first two are also located within the intron found in the 5' UTR. Six motifs recognized by SRY family HMG box DNA binding motifs (pheromone response motifs) 5'-CAAAG-3' (-289, -322, -571, -723, -772, and -1,372) were found in the respective region.

### Targeted deletion of GNA1

In order to delineate the regulatory targets of GNA1 signaling in *H. jecorina*, we first constructed a *gna1 *deletion strain by replacement of the coding region with the *H. jecorina pyr4 *gene conferring uridine prototrophy (Figure 1b). The resulting knockout strains showed a decreased (by roughly 70%) and clearly delayed sporulation compared to wild type and the *gna1*-retransformed strain. Cultivation of Δ*gna1 *on malt extract agar plates or plates containing Mandels-Andreotti medium with 1% (w/v) carboxymethylcellulose, glucose, or lactose as carbon source revealed only minor differences in hyphal apical extension rates in constant light and darkness, but on glycerol a reduction to approximately 70% was observed. Microscopic inspection of Δ*gna1 *also did not show the significant morphological aberrations as reported for the respective *N. crassa *mutant strain [21] such as hyperbranching or swellings in aerial hyphae (Additional file 1, supplementary figure S1). Additionally, unlike in *Neurospora*, we could not detect a positive influence of GNA1 on intracellular cAMP levels [22] in *H. jecorina *and hence adenylate cyclase activity, but we observed an increase of intracellular cAMP levels in the *gna1*-deletion mutant (105 ± 28 pmol/mg protein in the wild type versus 217 ± 40 pmol/mg protein in the mutant). This result corresponds well with data from the closely related *H. atroviridis *(anamorph *T. atroviride*) [34] and from *C. parasitica *[20], where deletion of the respective homologue also causes increased intracellular cAMP levels. This suggests that *H. jecorina *GNA1 represents a typical member of the adenylate cyclase inhibiting class I of G-protein α subunits.

### Generation of *H. jecorina *recombinant strains expressing constitutively activated GNA1

In order to create a *H. jecorina *strain which expresses a constitutively activated version of GNA1, a construct comprising the entire *gna1 *gene, but with the necessary alteration in the open reading frame was prepared. Therefore, a point mutation was introduced into GNA1, facilitating expression of a constitutively activated mutant protein with a single amino acid change within the GTPase domain at position 204 (Q_204 _to L; Figure 1c). Analogous mutations were previously reported to drastically reduce the intrinsic GTPase activity of Gα subunits, and to prevent returning of the respective G-protein to the inactive state [24,35-37]. Segers and Nuss [24] showed that these constitutively activated alleles are completely dominant over the wild-type allele in the case of ectopic integration. The *gna1*QL construct was used to transform the uridine auxotrophic *H. jecorina *strain TU-6. Constitutive activation caused a clear phenotype with delayed sporulation and reduced spore formation (less than 30%). In contrast to the *gna1*-deletion mutant, constitutive activation of GNA1 did not significantly alter cAMP levels (data not shown). Nevertheless, transcription of the mutated allele has been confirmed by reverse transcriptase polymerase chain reaction (RT-PCR) and subsequent sequencing of several random clones, which revealed the presence of the mutated sequence in addition to the wild type. Although our results clearly indicate presence and transcription of the altered allele as well as effects of this mutation, we do not have proof of abolishment of GTPase activity. Microscopic analysis as well as comparisons of the hyphal apical extension rates observed in mutant and wild-type strains on plates did not reveal severe morphological differences (data not shown).

### *gna1 *knockout impacts biomass formation

**Figure 2:**
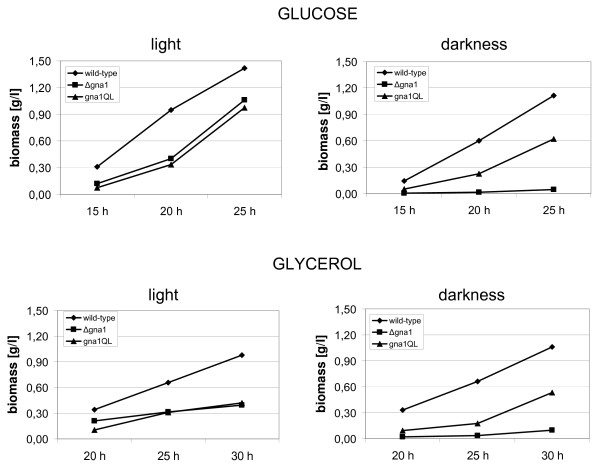
**Biomass accumulation upon growth on glucose or glycerol**. Strains were grown in liquid cultures on Mandels-Andreotti medium with 1% of glucose or glycerol as carbon source for the time indicated in constant darkness or constant light (1,800 lux). Biomass accumulation over time is shown for wild type, *gna1*QL and Δ*gna1*. Data from at least two independent experiments; the wild-type strain was included in every cultivation as a control. Standard deviations of the values given were below 10%.

Now having two strains with theoretically opposite characteristics with respect to GNA1 activity in hand, we could investigate the physiological roles attributable to GNA1 (indeed opposite effects in both strains) and distinguish them from those processes influenced by the Gβ and Gγ subunit (similar effects in these strains [23-25]). Because of the effect of deletion of *gna1 *on growth upon cultivation on agar plates on glycerol, biomass formation of Δ*gna1 *and *gna1*QL was measured upon growth in liquid culture using Mandels-Andreotti medium supplemented with 1% (w/v) of glucose or glycerol as carbon source, respectively. Upon growth in constant darkness, biomass accumulation of Δ*gna1 *was considerably decreased compared to the wild-type strain on both glucose and glycerol. In light however, biomass accumulation of Δ*gna1 *and *gna1*QL reached approximately equal levels, albeit they were significantly lower than in the wild-type strain (Figure 2). We conclude that GNA1 is involved in regulation of growth of *H. jecorina *in liquid culture predominantly in darkness, since these growth effects are not observed upon growth on solid medium or in light. Nevertheless, the major effect on growth seen in these experiments seems to be exerted via the influence of GNA1 on the G-protein β and γ subunits, since both deletion as well as constitutive activation of a Gα subunit cause increased levels of free Gβ-proteins and γ-proteins and hence similar effects on a given process.

### GNA1 influences reaction to conditions of stress

**Figure 3:**
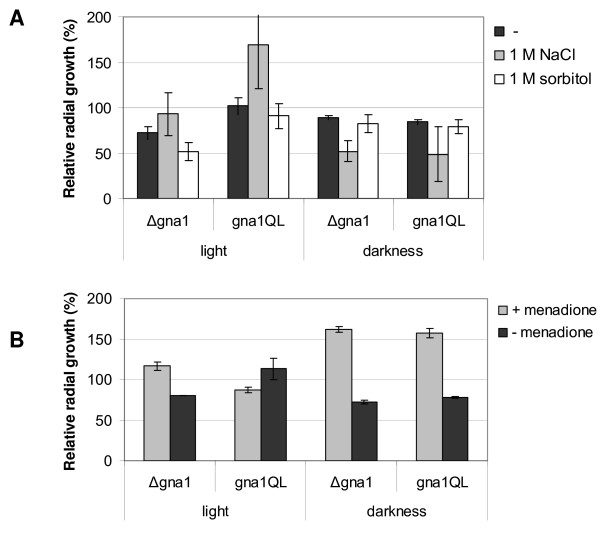
**Relative radial growth under conditions of stress**. Strains were grown on solid Mandels-Andreotti medium with 1% of glucose in constant light or constant darkness. Measurements of two different timepoints were combined. **(a) **Radial growth of *gna1*QL and Δ*gna1 *relative to the wild type under hyperosmotic conditions (1 M NaCl or 1 M sorbitol) in constant light or constant darkness. **(b) **Radial growth of *gna1*QL and Δ*gna1 *relative to the wild type under conditions of oxidative stress (that is, 0.25 mM menadione (M)).

Previous studies on the GNA1 orthologs *N. crassa *GNA-1 [21,25] and *C. parasitica *CPG-1 [24] revealed that these Gα subunits influence sensitivity to different kinds of stress. In *N. crassa*, deletion of *gna-1 *causes increased sensitivity to osmotic stress as compared to the wild type, while strains expressing a constitutively activated GNA-1 did not show such a defect. In *C. parasitica*, in contrast, deletion of *cpg-1 *caused enhanced growth on hypertonic media, whereas the respective constitutively activated mutants showed less tolerance to osmotic stress. The presence of several stress elements in the promoter of *H. jecorina gna1 *suggests a role of GNA1 in stress tolerance. However, contradicting effects in *N. crassa *and *C. parasitica *did not allow for prediction of the effect in *H. jecorina*. We therefore tested whether *H. jecorina *mutant strains deleted for *gna1 *or constitutive activation of GNA1 would show any differences to the wild type when grown on 1% glucose as carbon source with or without 1 M NaCl or 1 M sorbitol and with or without menadione, which causes oxidative stress. Figure 3 shows growth of the mutant strains relative to the wild-type strains under different stress conditions. Apical extension rate of the wild-type strain was 8.5 ± 0.7 mm/day in light or 7.8 ± 0.5 mm/day in darkness, respectively. The colony diameter of the wild type was decreased by 1 M NaCl by 92% in light and by 69% in darkness. Addition of 1 M sorbitol led to a reduction of radial growth by 46% in light and by 21% in darkness. Menadione led to a reduction in colony diameter in the wild type by 57% in light and by 83% in darkness. The results reveal that GNA1 only plays a minor role in tolerance of hyperosmotic conditions (Figure 3a), but shows interesting effects in dealing with oxidative stress.

Both the *gna1 *knockout as well as constitutive activation in most cases caused an increased inhibition by hyperosmotic conditions as compared to the wild type, albeit the variations are within standard deviations in some cases.

A different pattern was observed when these strains were analyzed for their response to oxidative stress (Figure 3b). In darkness both deletion of *gna1 *and constitutive activation of GNA1 caused enhanced growth in the presence of menadiaone as compared to the wild type. However, in light deletion of *gna1 *caused enhanced growth under conditions of oxidative stress, whereas constitutive activation of GNA1 resulted in decreased growth under these conditions.

Due to the similar direction of the effects caused by deletion or constitutive activation of GNA1, it appears that the βγ subunits are involved in the response to osmotic stress and that the (obviously minor) effect of GNA1 is caused by its influence on these subunits. The Gβ and γ subunits also seem to be largely responsible for the reaction to oxidative stress in darkness. However, in light the effect of GNA1 is most likely executed without the participation of the Gβ subunit.

### Transcription of *gna1 *is influenced by constitutive activation of its gene product

**Figure 4:**
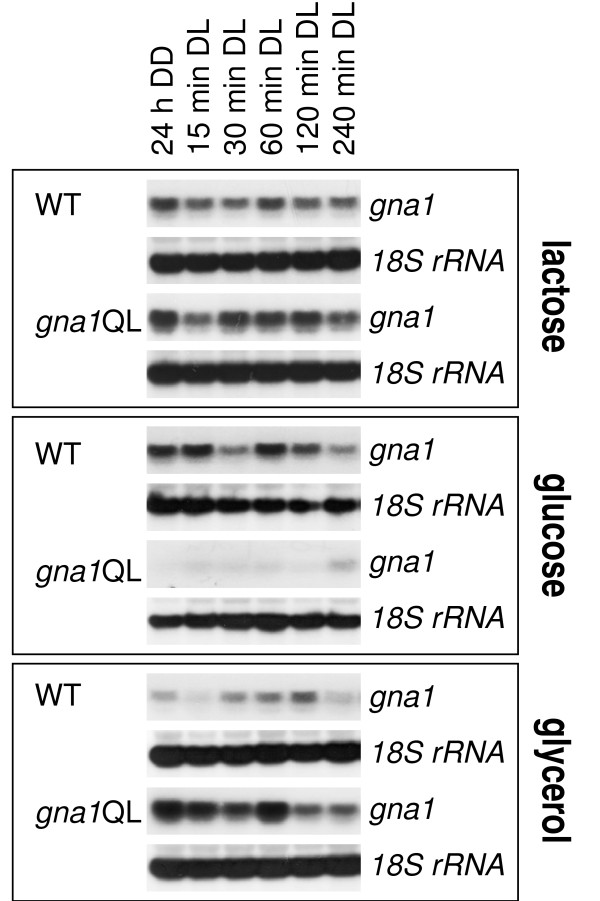
**Feedback regulation of *gna1 *transcription by constitutively activated GNA1**. Strains were grown in Mandels-Andreotti medium supplemented with 1% (w/v) lactose, glucose or glycerol as carbon source. Mycelia were precultivated for 24 h (lactose, glycerol) or 17 h (glucose) in darkness (DD) prior to exposure to light (DL; 1,800 lux, 25 μmol photons m^-2 ^s^-1^) for 15, 30, 60, 120 or 240 min. For northern blotting, 20 μg of total RNA were loaded per lane and [α^32^P]-radiolabeled PCR fragments of *gna1 *and *18S rRNA *were used as probes.

In order to determine the transcription characteristics of *gna1 *we analyzed wild-type and mutant strains on different carbon sources in darkness and after illumination. As the experiments described above indicated a light-dependent function of GNA1 the conditions chosen should also reveal a function in light response and adaptation. The availability of strains expressing a constitutively active GNA1 thereby allowed for the identification of a potential feedback mechanism, which would regulate transcription of *gna1 *and thus abundance of GNA1 (albeit posttranslational regulation must be considered) due to activation of the GNA1-related pathway (Figure 4). We observed that in contrast to transcription of the Gα subunit gene *gna3 *[13], transcription of *gna1 *is only marginally regulated in response to light in the wild type. On lactose, the transcript abundances and patterns of *gna1 *in the *gna1*QL strains resemble those of the wild-type strain. However, we found clear differences upon constitutive activation of GNA1 on glucose and glycerol. Thereby, transcript abundance of *gna1 *is strongly enhanced on glycerol in *gna1*QL as compared to the wild type (threefold to eightfold), but decreased (about fivefold) on glucose in *gna1*QL (Figure 4). Since the only difference in these strains to the wild-type strain is the presence of the additional allele (which is integrated ectopically) enabling expression of the constitutively active Gα subunit, we conclude that although transcribed, wild-type GNA1 normally is mainly present in its inactive state under these conditions and that these differences reflect the effect of activation of GNA1 in case of ligand binding to the receptor. These alterations also prove that the additional mutated *gna1 *allele is indeed expressed. Hence *gna1 *is subject to carbon source-dependent positive as well as negative regulation by activated GNA1.

### GNA1 regulates cellulase gene transcription in *H. jecorina*

**Figure 5:**
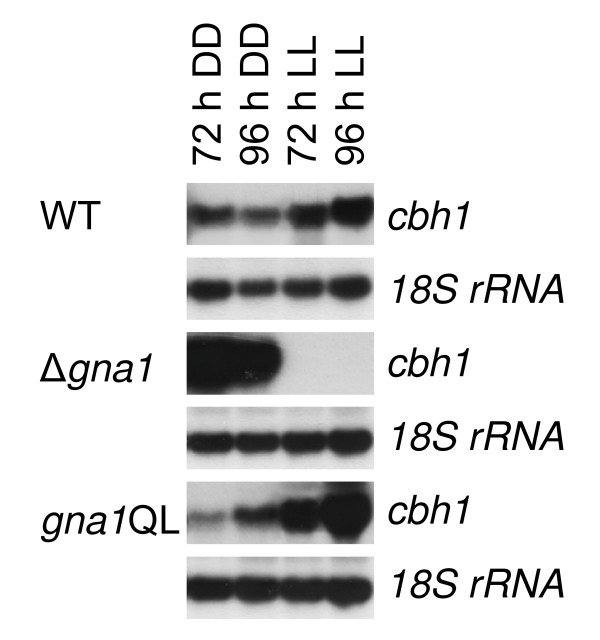
**Regulation of cellulase gene transcription by GNA1**. Transcription of the major cellulase *cbh1 *upon cultivation in constant light (LL) in the wild-type strain, *gna1*QL and in Δ*gna1 *upon growth in constant light or constant darkness with cellulose as carbon source. Mycelia were grown in Mandels-Andreotti medium supplemented with 1% microcrystalline cellulose as carbon source in constant light (1,800 lux, 25 μmol photons m^-2 ^s^-1^), indicated by LL, or constant darkness (DD) and harvested after 72 and 96 h. For northern blotting, 20 μg of total RNA were loaded per lane and [α^32^P]-radiolabeled PCR fragments of *cbh1 *and *18S rRNA *were used as probes.

Since the *C. parasitica *homologue of GNA1 (CPG-1) is essential for cellulase gene expression in this fungus [26], we tested whether GNA1 would exert a similar regulatory function in *H. jecorina*. The cellulase gene *cbh1*, encoding CEL7A, which makes up for 60% of the total cellulases secreted by *H. jecorina *was used to test this hypothesis. Most of the cellulases of *H. jecorina *have been shown to be coordinately induced by cellulose, lactose and sophorose in *H. jecorina *[4,38] and regulation of *cbh1 *can therefore be considered representative for cellulase regulation. Due to the reported effect of illumination on cellulase gene expression [11] we examined expression patterns either under constant illumination or constant darkness. Using constant illumination and 1% (w/v) microcrystalline cellulose as carbon source, *cbh1 *transcript accumulation was undetectable in the Δ*gna1 *mutant strain, while under the same conditions in constant darkness a strongly increased cellulase transcription (up to 10-fold compared to wild type) was detected (Figure 5). In the mutant strains expressing constitutively activated GNA1 we observed an increased accumulation of *cbh1 *transcripts on cellulose in light (about fivefold) and no major changes in darkness (Figure 5). Based on these findings we conclude that GNA1 is involved in regulation of cellulase gene expression on cellulose. This regulatory impact differs between cultivation in light or darkness.

### Constitutive activation of GNA1 does not overcome inducer dependence of cellulase formation in *H. jecorina*

The influence of GNA1 on transcription of *cbh1 *could in theory be interpreted by activation of GNA1 after binding of the cellulase inducer to the G-protein coupled receptor associated with GNA1. Consequently, GNA1 would transmit the cellulose signal from outside the cell to the cellulase transcription machinery. If this were the case, strains bearing the constitutively activated *gna1 *allele should also be capable of forming cellulases in the absence of their inducer. To test this possibility, we grew these strains on glycerol or glucose as carbon source, and examined cellulase transcription. On glycerol, cellulase expression can be induced by the addition of for example the strong inducer sophorose, whereas glucose represses the induction of cellulase gene expression by inducer exclusion (for a review see [5]). However, no *cbh1 *transcript could be detected under these conditions, neither in darkness nor after several hours of illumination (data not shown). Thus the constitutive activation of GNA1 does not result in inducer-independent cellulase gene transcription. Consequently, it could be concluded that GNA1 transmits a fundamental signal for cellulase gene expression, but this signal is not the sole determinant for their induction. Considering there are 34 G-protein coupled receptors and only 3 Gα subunits in the genome of *H. jecorina *[27] the GNA1 signal(s) is/are important for cellulase regulation but not sufficient for their induction.

### GNA1 impacts regulation of the hydrophobin genes *hfb1 *and *hfb2*

Previous studies on CPG-1, the *C. parasitica *homologue of GNA1, revealed that this G-protein α subunit suppresses transcript accumulation of the hydrophobin cryparin (*crp*) [24]. We therefore tested whether GNA1 has an influence on the formation of the two best characterized [39,40] class II hydrophobins of *H. jecorina*, *hfb1 *and *hfb2*. Since the transcription of hydrophobins is also influenced by illumination [40], these experiments were again performed in the presence and absence of light.

**Figure 6:**
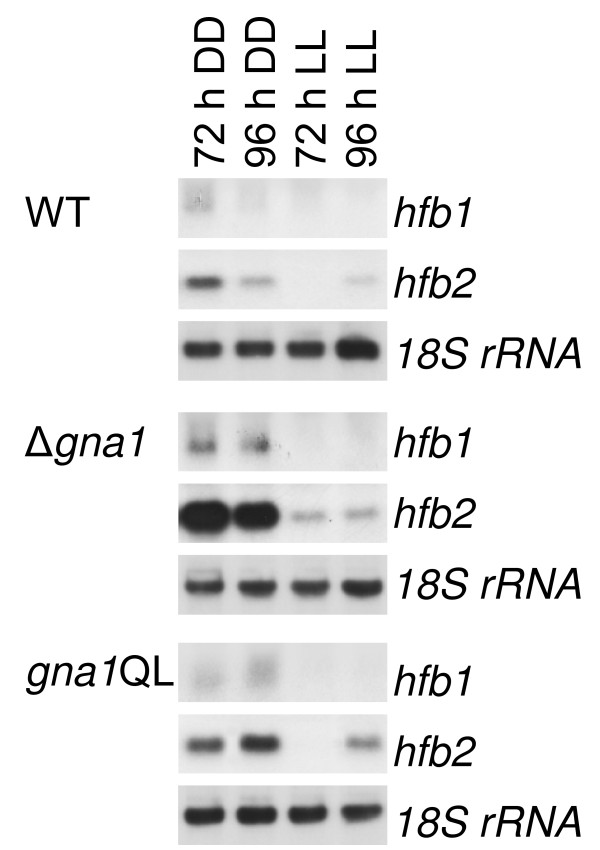
**Regulation of the hydrophobin genes *hfb1 *and *hfb2 *by GNA1**. Mycelia were grown in Mandels-Andreotti medium supplemented with 1% (w/v) microcrystalline cellulose as carbon source in constant light (1,800 lux, 25 μmol photons m^-2 ^s^-1^), indicated by LL, or constant darkness (DD) and harvested after 72 and 96 h. For northern blotting, 20 μg of total RNA were loaded per lane and [α^32^P]-radiolabeled PCR fragments of *hfb1, hfb2 *and *18S rRNA *were used as probes.

The results are shown in Figure 6: *hfb1 *and *hfb2 *transcripts are found to be more abundant in constant darkness than in constant light upon cultivation on cellulose. Both genes are upregulated in the Δ*gna1 *strain in darkness (up to 10-fold for *hfb2*), whereas GNA1 had hardly any influence in the presence of light. Constitutive activation of GNA1 did not have a considerable effect on transcription of *hfb1 *and *hfb2*. Thus GNA1 seems to be active under the conditions tested and most likely establishes an equilibrium upon activation, which becomes imbalanced when *gna1 *is lacking, but which cannot be altered considerably by expression of additional, constitutively active GNA1.

## Discussion

Elucidation of the roles of heterotrimeric G-protein pathways in various important processes in fungi has been subject to intensive investigation in recent years [41]. Consequently, many different functions specifically exerted by the Gα subunit as well as such exerted via the effect of deletion or constitutive activation of the Gα subunit on the β and γ subunit functions [23-25] for these proteins are already known [14,41]. These detailed studies also revealed that despite considerable sequence similarity among Gα subunits, their functions in some cases show distinct variations between species. In this study we report on the characterization of a G-protein α subunit encoding gene (*gna1*) from the ascomycete *Hypocrea jecorina *(anamorph *Trichoderma reesei*). For the first time in fungi we show here a detailed analysis of the effect of a class I Gα subunit under controlled light conditions. As was previously reported for orthologs in other fungi, we observed an influence of GNA1 on sporulation efficiency [34,42] as well as biomass formation [21,43]. Interestingly, the influence on biomass formation was dependent on the light status. This suggested that GNA1 might relate a carbon source-dependent signal, the physiological significance of which differs between light and darkness. The severe growth defect of the *gna1 *deletion compared to the same mutation in other fungi [20,21,34] was only observed in darkness and only in liquid culture.

Since the effect of the deletion or constitutive activation of GNA1 does not point in different directions (decreased biomass formation in both cases) in light, it most likely involves the function of the Gβ subunit. Similarly, Yang *et al*. [44] showed that the *N. crassa *G-protein β subunit GNB-1 directly regulates apical extension rate and mass accumulation. A light-dependent function of *N. crassa *GNA-1 in this respect has also been reported [21]. While our results do not allow for a clear determination of the contribution of GNA1 to this mechanism, the finding that deletion of *gna1 *results in a severe growth defect in darkness both on glucose and glycerol indicates that growth is likely to be also regulated by GNA1 and not only by its influence on the G-protein β and γ subunits.

*H. atroviridis *strains deleted for the GNA1 homologue TGA1 show drastically reduced growth and form compact upraised colonies. In addition, continuous, light-independent conidiation was observed with these strains [34]. Rocha-Ramirez *et al*. [42] reported that *H. atroviridis tga1 *overexpressing strains and strains expressing constitutively activated TGA1 exhibit a fluffy phenotype and no alteration in growth compared to the wild type but inhibited sporulation. None of the overexpression strains produced a conidiation ring after a light pulse. Gα antisense mutant strains were characterized by the formation of rather dense and small colonies, continuous sporulation independent of light stimulation and the production of a yellowish pigment [42]. These results are in striking contrast to our findings for *H. jecorina*, which showed only minor growth defects on solid medium caused by deletion of *gna1 *compared to *H. atroviridis*, which suggests at least in part different output targets of the GNA1 signal in these two fungi.

The main objective of this study was to investigate whether *gna1 *would be involved in the transduction of cellulase inducing signals, a regulatory target for heterotrimeric G-protein signaling, as recently also shown for *H. jecorina *GNA3 [13]. First hints as to an involvement of G-protein-mediated signal transduction in regulation of cellulase gene expression were provided by [26] in describing the involvement of CPG-1 in *C. parasitica*. This G-protein α subunit is 98% identical to GNA1 in *H. jecorina *and is involved in cellulose-mediated induction of the biosynthesis of cellulases. In accordance with these results it could be speculated that constitutive activation of GNA1 might lead to inducer independent cellulase transcription. Additionally, cAMP levels, which are influenced by GNA-1 in *N. crassa *[22], but only moderately in *H. jecorina*, have been shown to modulate cellulase gene expression [45] as does the light status during cultivation [11]. Indeed, we show that *H. jecorina *GNA1 is an important member of the signaling cascade targeting cellulase regulation and that obviously the light status is important for the effect (and thus relevance) of its receptor signal along with the carbon source available. However, constitutive activation of GNA1 did not cause cellulase gene expression in the absence of an inducer. We conclude that GNA1 transmits a signal, which is essential for cellulase transcription on cellulose only in light, but has a negative effect in darkness, which however does not lead to a decrease below wild-type levels upon constitutive activation of GNA1. Hence further factors are likely to establish and maintain certain cellulase levels.

At the present stage we cannot specify whether the light response pathway acts directly on GNA1 or on signaling components downstream of GNA1. Nevertheless, a regulation of transcription of *gna1 *by the white collar complex (WCC) comprising the *H. jecorina *orthologs of *N. crassa *WC-1 and WC-2 (BLR1 and BLR2) is unlikely since no significant light regulation was observed for *gna1*. However, an involvement of these photoreceptors in regulating activity or stability of GNA1 cannot be excluded. Constitutive activation of GNA3, another Gα subunit also does not enable inducer independent cellulase expression [13]. Intriguingly, a similarly strong effect on cellulase gene transcription as upon deletion of *gna1 *in darkness is seen for constitutive activation of GNA3 in light. An interconnection of the roles of these Gα subunits with respect to the light status as well as the involvement of BLR1 and BLR2 in this process is therefore currently subject to further investigation. These results indicate that higher order regulatory factors must be present to facilitate both basic levels as well as fine-tuned adaptation to the environment, which is reflected by an obviously restricted effect of GNA1 in some cases. Hence both Gα subunits relate a signal, the relevance of which depends on the light status, but is only modulary instead of essential for cellulase gene expression under most conditions. Additionally, the presence of 34 G-protein coupled receptors, but only 3 Gα subunits in the genome of *H. jecorina *[27], suggests that GNA1 and possibly GNA3 transmit several signals and thus integrate these environmental cues to a defined message in the signaling cascade.

Our ongoing studies on heterotrimeric G-protein signaling suggest a regulatory interrelationship between *gna1 *and *gna3 *as well as an interconnection to the light response pathway (data not shown), which warrants further detailed investigations. It seems that such a sensing mechanism needs additional regulatory levels. Consequently, while our data clearly show that GNA1 is involved in regulation of cellulase gene expression, a general role in the respective induction process was not observed. GNA1 is likely to only represent one of several key components necessary to initiate cellulase gene expression and the precise nature of the signal GNA1 transmits, remains to be determined.

Due to the only moderate influence of GNA1 on cAMP levels, the considerable effect on cellulase gene expression upon deletion of *gna1 *is unlikely to be a consequence of this function. Rather, it can be expected that GNA1 is one constituent of a group of signaling components establishing a steady state equilibrium, which becomes unbalanced upon deletion of GNA1.

The surprising impact of constitutive activation of GNA1 on transcription of *gna1 *could be interpreted as a carbon source-dependent feedback cycle, which can define the significance of the GNA1 signal(s) under certain conditions by regulating its abundance, although a potential effect of both stability and activation of GNA1 must be kept in mind. Such a mechanism would necessitate that one target of the GNA1 signal transduction pathway would be a transcription factor binding to the *gna1 *promoter. The fact that this feedback is different on different carbon sources suggests that binding, activation (for example by phosphorylation) and/or stability (for example by ubiquitinylation) of this transcription factor are dependent on the carbon source. Such a feedback cycle could determine the relevance of the respective signal under the current environmental (nutritional) conditions. By adjusting transcription of GNA1, rapid and appropriate response to these changing conditions is facilitated, once the receptor becomes activated. However, additional studies will be necessary to confirm this hypothesis and to elucidate the members of such a mechanism and their functions.

Segers and Nuss [24] suggested a surveillance system, which would modulate the stability of a subunit in response to a mutation or absence of a certain component of the heterotrimeric G-protein signaling machinery. Considering our data this hypothesis could be modified to integrate environmental signals into the regulation of stability, and additionally transcription efficiency, of a certain subunit. The postulated feedback regulation of *gna1 *and especially the light-dependent role in cellulase transcription thus supports the hypothesis of a sophisticated fine-tuning mechanism for crosstalk of light response and carbon source signal transduction as already suggested earlier [11].

Regulation of a hydrophobin gene by a Gα subunit was first shown for the GNA1 homologue of *C. parasitica *[24]. In support of this result, we also observed different wetability of mycelia in strains expressing constitutively activated GNA1 or deleted for *gna1*. Hydrophobins have been shown to be regulated due to the developmental stage and in response to numerous environmental cues such as the nature or availability of nutrients and light not only in *H. jecorina *[39,40], but also in several other fungi [46-49]. Because of the possible use of hydrophobins in industrial applications [47,50] these findings can also contribute to strain improvement and optimization of the respective biotechnological processes.

Analysis of transcription of the two class II hydrophobin genes *hfb1 *and *hfb2 *of *H. jecorina *suggest that GNA1 negatively regulates both genes. Although *hfb1 *and *hfb2 *have been shown to be involved in regulation of hyphal development (*hfb1*) and sporulation (*hfb2*) [39] our data on these characteristics in light and darkness for constitutive activation or deletion of *gna1 *do not support the hypothesis that the phenotype of these mutants could be explained by the effect of GNA1 on the hydrophobin genes. Additionally, GNA1 appears not to be solely responsible for (down)regulation of hydrophobin expression since constitutive activation does not result in decreased transcription below wild-type levels. As is the case with regulation of cellulase gene expression, GNA1 delivers an important yet not essential signal for regulation of *hfb1 *and *hfb2*.

## Conclusion

In summary, GNA1 is suggested to be involved in appropriating available resources to be used in biomass formation, biosynthesis of hydrolytic enzymes or promotion/obstruction of another energy-consuming process of higher/lower priority under the current environmental conditions. One important environmental cue thereby is the presence of light, which now has been shown to influence two Gα subunits in *H. jecorina*. Hence GNA1 (in addition to GNA3) is likely to be important for adaptation to the day/night cycle. This hypothesis is mainly supported by the indications of an involvement of GNA1 or the Gβγ complex, respectively, in a light-dependent manner for different processes, but also by the recent findings of a light-dependent function of GNA3 [13]. Carbon source-dependent feedback regulation would be one logical junction for such modulations. Autoregulation of Gα subunits has been suggested in mammals [51] and positive as well as negative feedback regulation via downstream effectors of Gα subunits has been shown in *Saccharomyces cerevisiae *[52]. Moreover, certain feedback regulation via the Gβγ complex cannot be excluded in *N. crassa *[44]. We postulate that GNA1 is likely to contact different G-protein coupled receptors and that the respective signaling pathway is interconnected with other signaling and regulatory pathways, which limit the effect of the GNA1 signal.

## Methods

### Microbial strains and culture conditions

The uridine auxotrophic *H. jecorina *(*T. reesei*) strain TU-6 (ATCC MYA-256; Δ*pyr4*; [53]) was used for construction of the recombinant strains described in this study and maintained on malt extract agar supplemented with 10 mM uridine (Sigma-Aldrich, Madison, WI, USA). The strain TU-6/*pyr4+ *(Δ*pyr4, pyr4*^+^) retransformed with the *pyr4 *fragment of the vector pFG1 [53] was maintained on malt extract agar. This strain was used as control strain reflecting wild-type (WT) conditions in all experiments. The recombinant mutant strains *gna1*QL1 and *gna1*QL2 (Δ*pyr4; gna1*Q204L^+^::*pyr4*^+^) prepared throughout this study were maintained on selective minimal medium (1 g/l MgSO_4_.7H_2_O, 10 g/l KH_2_PO_4 _1%, 6 g/l (NH_4_)_2_SO_4 _3 g/l trisodium citrate. 2H_2_O, 10 g/l glucose, 20 ml/l 50 × trace elements solution (0.25 g/l FeSO_4_.7H_2_O, 0.07 g/l ZnSO_4_.2H_2_O, 0.1 g/l CoCl_2_.6H_2_O, 0.085 g/l MnSO_4_.H_2_O), agar-agar 2% (w/v), all chemicals by Merck, Darmstadt, Germany). Strains were grown in 1-l Erlenmeyer flasks at 28°C on a rotary shaker (200 rpm) in 200 ml of minimal medium as described by Mandels and Andreotti [54] supplemented with 0.1% (w/v) peptone to induce germination and 1% (w/v) of carbon source, as indicated at the respective experiments (that is, microcrystalline cellulose (# 14204, SERVA, Heidelberg, Germany), lactose, D-glucose, carboxymethylcellulose or glycerol (Merck, Germany)). Approximately 10^8 ^conidia/l (final concentration) were used as inoculum.

Strains were grown under the conditions specified in the legends in light (LL, 25 μmol photons m^-2 ^s^-1^; 1,800 lux, daylight lamps, white light), in constant darkness (DD) or transferred from darkness to light (DL) (that is, preculture in constant darkness for 24 h (glycerol, lactose) or 17 h (glucose) and illumination for up to 4 h). In case of dark grown cultures harvesting of the mycelia was performed under red safety light, which was shown to have no effect on the fungus [11].

*Escherichia coli *JM109 [55] was used for the propagation of vectors. Standard LB medium [56] was used for cultivations of *E. coli*.

Media for stress response experiments were prepared by adding 1 M NaCl or 1 M sorbitol to Mandels-Andreotti medium with 1% (w/v) of glucose as carbon source prior to autoclaving. Menadione sodium bisulfite (Sigma-Aldrich), which generates superoxide anion radicals [57], was added to a final concentration of 0.25 mM from an aqueous stock solution to autoclaved Mandels-Andreotti medium with 1% of glucose (w/v) as carbon source. Plates were inoculated at 28°C in constant light (1,800 lux) or constant darkness. Results were combined from two independent experiments with three replicates each.

### DNA and RNA manipulations

Fungal mycelia were harvested by filtration, frozen and ground in liquid nitrogen. Genomic DNA was isolated as described previously [58]. For northern blots, total RNA was isolated by the guanidinium/phenol procedure [58,59]. Standard methods [60] were used for electrophoresis, blotting and hybridization of nucleic acids. The probes used for hybridization were generated by PCR. Sequences of the respective primers used are given in Table 1. The wild-type strain was included in every cultivation to allow for representative evaluation of the data obtained. The data shown originate from at least two independent experiments yielding consistent results. Densitometric scanning of autoradiograms was performed using the BioRad GS-800 (BioRad, Madison, WI, USA) calibrated densitometer. Measurements were normalized to 18S rRNA signals.

**Table 1:** Oligonucleotides used in this study

**Name**	**Sequence**	**Application**
cbh1SF	5'-TCGGCCTGCACTCTCCAATCC-3'	PCR fragments used as probes for northern blots
cbh1SR	5'-TGGAGTCCAGCCACAGCATG-3'	
18SRF	5'-GGTGGAGTGATTTGTCTG-3'	
18SRR	5'-CTTACTAGGGATTCCTCG-3'	
hfb1F	5'-CTGAACACCTCCAGTCAAC-3'	
hfb1R	5'-TATTGGAGAATAAGAATGGC-3'	
hfb2F	5'-TCAAGATGCAGTTCTTCG-3'	
hfb2R	5'-CATTGCTTTAGAAGGTGC-3'	

gna1aa5F	5'-ACACGAACGCCCAAATCTG-3'	Construction of vector pBgna1Q204L
gna1aa5R	5'-CTTTCGCTCAGATCGC**T**G-3'	
gna1aa3F	5'-GCC**A**GCGATCTGAGCGAAAGAAG-3'	
gna1aa3R	5'-GGGCGACGGCACATTAACATAG-3'	
gna1aa5NF	5'-atgtcgacCAAATCTGCGTCTTCCACAC-3'	
gna1aa3NR	5'-atgggcccCGGCACATTAACATAGCTTG-3'	

gna1F	5'-TAAGCGAAGGTTCTCCTG-3'	cDNA amplification
gna1R	5'-ATTGGCATTTCATTCCTC-3'	

gna1D5F	5'-atgggcccCATCTCCACAGTTTCCAGG-3'	Construction of vector pDELgna1
gna1D5R	5'-atgtcgacTGTTGGCGACTGTGAATG-3'	
gna1D3F	5'-atggatccTTAGACGACTTGTTGATTGTTG-3'	
gna1D3R	5'-attctagaCCCAATGCTTATAGAACATC-3'	

Mutated bases are given in bold, artificially introduced restriction sites are given in lower case letters.

PCR = polymerase chain reaction.

### Preparation of *gna1 *deletion strains and retransformation

The *gna1 *gene was identified in the genome sequence database of *H. jecorina *([28]; Figure 1a) by tblastn searches with the well characterized Gα subunits CPG-1 of *C. parasitica *and GNA-1 of *N. crassa *and its sequence was used to design primers for amplification and cloning. Sequences of all primers used throughout this study are given in Table 1.

To obtain a *gna1 *deletion mutant, *H. jecorina *TU-6 was transformed with plasmid pDELgna1 which contains the complete open reading frame of *gna1 *replaced by the *pyr4 *gene which confers uridine prototrophy to the auxotrophic strain TU-6 [53] (Figure 1b). This vector was constructed as follows: a 1,013 bp fragment of the 5'-flanking sequence of *gna1 *was amplified by PCR using primers gna1D5F and gna1D5R, the amplicons cleaved with *Apa*I and *Sal*I (all restriction enzymes by Fermentas, Vilnius, Lithuania) and a 982 bp fragment of the 3'-flanking region of *gna1 *was amplified by PCR using primers gna1D3F and gna1D3R and digested with *Eco*RI-*Xba*I. These fragments were subsequently ligated into the respective restriction sites of pBluescript SK+ and the *pyr4 *marker cassette, excised from pFG1 [53], was inserted into the *Sal*I site resulting in the deletion vector pDELgna1. The transformation cassette was excised from pDELgna1 by restriction digestion with *Apa*I and *Xba*I and 10 μg were used for transformation of *H. jecorina *TU-6 [53]. Transformants were selected on selective minimal medium as described above. Fungal DNA was isolated from transformants using standard protocols and subjected to PCR using primers gna1aa5F and gna1aa3R, where in the deletion mutant appearance of a 4,153 bp instead of the 2,645 bp wild-type band reflected successful deletion. Southern blot analysis was used to confirm this result (Additional file 2, supplementary figure S2 A and B).

Complementation of Δ*gna1 *with the *gna1 *wild-type gene was achieved by cotransformation of a PCR fragment amplified with primers gna1D5F and gna1D3R comprising the entire *gna1 *gene and the hygromycin marker cassette [61]. Successful complementation was confirmed by PCR. Wild-type behavior was restored in the retransformant.

### Construction of a *H. jecorina *gna1QL strain

For construction of a modified copy for expression of a constitutively activated version of GNA1, an overlap extension mutagenesis approach was used. This procedure resulted in the single amino acid modification Q204L, which has been reported to impair the intrinsic GTPase activity of this protein [24,35,36]. Four oligonucleotides (Table 1) were designed based on the genomic sequence to generate the desired mutation in three steps (Figure 1c). In a first PCR, primer gna1aa5F and gna1aa5R were used to amplify the 1,202 bp 5' region. In a second PCR, primers gna1aa3F and gna1aa3R were used to amplify the 1,464 bp 3' region. Afterwards, these fragments were purified, mixed and used as template in a third PCR in order to obtain a 2,645 bp fragment bearing the intended mutation as well as flanking regions 3' and 5' by using the nested primers gna1aa5NF and gna1aa3NR, which in addition contains an artificial *Apa*I and a *Sal*I restriction site to facilitate cloning. The PCR product was cloned into *Apa*I-*Sal*I sites of pBluescript SK+ (Stratagene, La Jolla, CA, USA) thereby generating pBgna1Q204L. The *gna1 *coding region was completely sequenced to ensure that only the desired mutations had been introduced. After linearization by *Apa*I, 10 μg of this fragment were used to transform protoplasts of *H. jecorina *TU-6 in cotransformation with 2 μg of a 2.7 kb *Sal*I *pyr4 *fragment excised from vector pFG1 [53] conferring uridine prototrophy. Transformants were selected on minimal medium as described above. Stable transformants were obtained by at least two rounds of single spore isolation or three rounds of transfers to selection medium lacking uridine in case of non-sporulating mutants. Integration into the *H. jecorina *genome and copy number was analyzed by Southern blotting, using *Bam*HI and *Hin*dIII, which cut within *gna1 *and additionally within the vector backbone, thereby showing the presence of the cassette by the appearance of an additional 1.5 kb band (Additional file 1, supplementary figure S1 C). The presence of the 2,198 bp and 5,201 bp wild-type bands in both wild-type and mutant strains confirmed ectopic integration. Two strains (*gna1*QL1 and *gna1*QL2) bearing one or two ectopically integrated copies of the constitutively activated *gna1*-allele were chosen for further analysis. Consistent results were obtained for both strains in the following experiments. Actual expression of the mutated allele was checked by RT-PCR and sequencing of the cloned transcripts and is also reflected by the divergent effects of the presence of this allele on different carbon sources.

### cDNA preparation and RT-PCR

Total RNA was treated with DNase I (Fermentas) to remove contaminating chromosomal DNA. cDNA was prepared from total RNA using the Clontech Creator SMART cDNA library construction kit (Takara Bio, Tokyo, Japan). GoTaq Polymerase (Promega, Madison, WI, USA) was used for PCR amplification of the *gna1 *cDNA.

### Measurement of intracellular cAMP levels

Mycelia grown on agar plates with Mandels-Andreotti medium with carboxymethylcellulose (1%, w/v) as carbon source, covered with cellophane, for 72 h in constant light were ground in liquid nitrogen to a fine powder, weighed, and suspended in 10 ml/g mycelium of 0.1 M HCl. Inoculation of the plates was performed using a 3 mm diameter agar slice with sporulated mycelium of the respective strain, which was placed in the middle of the plate. The samples were centrifuged at 600 *g *at 22°C for 10 min, and the cAMP concentration measured with the Direct cAMP enzyme immunoassay kit (Sigma-Aldrich) according to the manufacturer's instructions. cAMP levels were related to the protein content of the sample and combined from two independent experiments.

## Authors' contributions

CS participated in drafting the manuscript and performed retransformation of the *gna1*-deletion strain. GG worked on deletion of *gna1 *and performed the northern analyses. RS created the gna1QL strain. AS contributed analyses of biomass formation and growth under stress conditions. CPK participated in drafting the manuscript and conception of the study. MS conceived of the study, interpreted results and wrote the manuscript.

## Supplementary Material

Additional file 1 **Supplementary Figure S1**. Microscopic observation of growth of Δ*gna1 *compared to wild-type. Click here for file

Additional file 2 **Supplementary Figure S2**. Confirmation of deletion of gna1 or ectopic integation of an additional allele for expression of constitutively activated GNA1. Click here for file
