# Supplementary material for: Light-dependent roles of the G-protein α subunit GNA1 of Hypocrea jecorina (anamorph Trichoderma reesei)
Source: BMC Biol. 2009 Sep 3;7:58. doi: 10.1186/1741-7007-7-58 (PMC2749820; doi:10.1186/1741-7007-7-58)
Supplement: Additional file 1 — Supplementary Figure S1. Microscopic observation of growth of Δgna1 compared to wild-type. [file 1741-7007-7-58-S1.doc]

Supplementary figure S1.

**Microscopic observation of growth of Δ*gna1* compared to wild-type**.

No major defects in hyphal morphology have been observed in Δ*gna1* compared to wild-type upon growth on solid Mandels Andreotti medium with 1 % glycerol (w/v) as carbon source in light (1800 lux, 25 µmol photons m^-2^ s^-1^) or darkness.
