# Supplementary material for: Light-dependent roles of the G-protein α subunit GNA1 of Hypocrea jecorina (anamorph Trichoderma reesei)
Source: BMC Biol. 2009 Sep 3;7:58. doi: 10.1186/1741-7007-7-58 (PMC2749820; doi:10.1186/1741-7007-7-58)
Supplement: Additional file 2 — Supplementary Figure S2. Confirmation of deletion of gna1 or ectopic integation of an additional allele for expression of constitutively activated GNA1. [file 1741-7007-7-58-S2.doc]

Supplementary figure S1.

**Confirmation of deletion of gna1 or ectopic integation of an additional allele for expression of constitutively activated GNA1.**

(A) Verification of *gna1* deletion via PCR amplification. As expected, a larger fragment (4700 bp; M...kb ladder) was obtained in case of the *Δgna1* mutant strain compared to the fragment amplified from wild-type (WT) or negative mutant strains (MS1 and MS2).

(B) Southern blot analysis for the detection of *Δgna1* mutant strains. Genomic DNA was digested with *EcoR*I and probed with an α-^32^P radiolabelled PCR fragment amplified with primers gna1D5F and gna1D3R (see table1). In case of the wild-type (WT) bands at 7531 bp, 5458 bp, 1055 bp and 493 bp were expected. Positive *Δgna1* mutant strains should show bands at 7531 bp, 5838 bp and 517 bp. For comparison a negative mutant strain (MS1) is shown.

(C) Southern blot analysis of ectopic integration of the mutated *gna1* allele for the expression of constitutively active GNA1. Genomic DNA was digested with *Hind*III/*BamH*I and probed with a 1030 bp α-^32^P radiolabelled PCR fragment amplified with primers gna1aa3F and gna1aa3R (see table 1). For the wild-type (WT) bands at 2198 bp as well as 5207 bp were expected. An additional band at 1500 bp is characteristic for positive mutant strains (QL1 and QL2) containing the transformation cassette.
